# Supplementary material for: Dual role of an essential HtrA2/Omi protease in the human malaria parasite: Maintenance of mitochondrial homeostasis and induction of apoptosis-like cell death under cellular stress
Source: PLoS Pathog. 2022 Oct 28;18(10):e1010932. doi: 10.1371/journal.ppat.1010932 (PMC9645662; doi:10.1371/journal.ppat.1010932)
Supplement: S1 Method — (DOCX) [file ppat.1010932.s001.docx]

**Supplementary Method:**

**Identification and sequence analysis of an HtrA2 homologue in *P. falciparum*, *Pf*HtrA2**

A sequence search in *P. falciparum* genome database identified a putative homolog of HtrA2 (Gene ID: PF3D7_0812200). The *P. falciparum* HtrA2 (*Pf*HtrA2) is a 404 amino acid long protein; sequence homology analysis showed that *Pf*HtrA2 has 30% amino acid identity to its homologs in *E. coli* (E-value: 4e^-20^) and 26% amino acid identity to *Homo sapiens* HtrA2/Omi (E-value: 7e^-11^) respectively. Protein sequence analysis using Pfam database showed that *Pf*HtrA2 contains a trypsin like protease domain (138-283 aa; E-value: 1.2e^-15^) and a C-terminal PDZ domain (316-404 aa; E-value: 7.9e^-08^); however, it lacks any transmembrane domain which are found in human HtrA2 (Fig. 1A). ClustalW multiple sequence alignment of the protease domain with that of human and bacterial homologs revealed that the catalytic residues, histidine, aspartic acid, and serine, are conserved in *Pf*HtrA2 (Fig. S1). A search for signal sequences using MitoProt II and PlasMit tools showed presence of a mitochondrial localisation signal at the N-terminus of *Pf*HtrA2 (1-28 aa) (Score 0.77). From genome database different homologs of HtrA2, *P. knowlesi* (PKNH_1428400), *P. vivax* (PVP01_1428000), *P. chabaudi* (PCHAS_1427100), *P. ovale* (PocGH01_14035300), *P. malariae* (PmUG01_14044000) and *P. berghei* (PBANKA_1425300), are retrieved and multiple sequence analysis of protein sequences of these genes showed that *Pf*HtrA2 is highly conserved in different *Plasmodium* species showing consensus level >70% (Fig. S2).

**Cloning, Expression and purification of recombinant proteins and generation of polyclonal anti-sera:** Fragment of *Pf*HtrA2 corresponding to trypsin like protease domain (135 aa-290 aa) was PCR amplified from total cDNA of *P. falciparum* 3D7, using 959A-960A primers and cloned into pET28a (Novagen) expression vector using *Bam*H1 and *Xho*1 restriction sites. Fragment of *Pf*HtrA2 corresponding to C-terminal PDZ domain (287 aa-403aa) was PCR amplified from total using 1461A-1462A primers and cloned into pET32a (Novagen) expression vector using *Bam*H1 and *Xho*1 restriction sites. Recombinant proteins, *Pf*HtrA2-protease His-tagged at the N-terminus and *Pf*HtrA2-PDZ His- and MBP-tagged, were expressed using *Escherichia coli* BL21(DE3) cells. Briefly, expression of the recombinant protein, was induced with 1 mM isopropylthio-β-D-galactoside (IPTG) at OD_600 nm_ for 4 h, at 37°C. Recombinant protein was purified from cytosolic fractions of the *E. coli* cell lysate by affinity chromatography using Ni^2+^ NTA resins.

Purified recombinant protein (250 μg) was injected into New Zealand white rabbit in emulsion with complete Freund’s adjuvant (Sigma, USA) on 0 day, followed by two boosters in incomplete Freund’s adjuvant on day 28 and 49. The rabbit sera were collected on day 56. The antibody titter in the serum samples was quantified using ELISA.

**Protease activity assay, enzyme kinetics and inhibition analysis:** Protease activity assay of recombinant protein was standardized using different fluorometric substrates (GGL-AMC, LLVY-AMC, Z-FR-AMC, Z-LRGG-AMC, Z-LR-AMC, and Z-AFK-AMC) at final concentration of 1µM in 0.1M sodium Phosphate buffer containing 1mM DTT. The release of free AMC was monitored as change in fluorescence units (excitation 355 nm; emission 460 nm) for 30-120 min at 37° C using a Victor-X3 Multi Label reader (Perkin-Elmer). The assay was performed in presence and absence of HtrA2 specific inhibitor ucf-101 at varying concentration of ucf-101. The *K*m and *V*max values were obtained using Graph Pad Prism V5.0 software.
