# Supplementary material for: Dual role of an essential HtrA2/Omi protease in the human malaria parasite: Maintenance of mitochondrial homeostasis and induction of apoptosis-like cell death under cellular stress
Source: PLoS Pathog. 2022 Oct 28;18(10):e1010932. doi: 10.1371/journal.ppat.1010932 (PMC9645662; doi:10.1371/journal.ppat.1010932)
Supplement: S1 Table — (DOCX) [file ppat.1010932.s002.docx]

## Table S1: List of primers used in the study

| Primer | **Sequence** |
| --- | --- |
| 959A | GCGGATCCTTGGGATCTGGATTTATTTAC |
| 960A | GCCTCGAGCATGCCCACAAGATTGCCATG |
| 1236A | TACGGATACGCATAATCGG |
| 1237A | CAGGCTTTACACTTTATGCTT |
| 1379A | GCAGATCTGCATACCATAATGTTGATAC |
| 1380A | GCCTGCAGCATATTTGACCTAAAACATTAAAATAA |
| 1461A | GCGGATCCACCATGGAAGATAATTATGGATTGGCTTTGCC |
| 1462A | GCGCGGCCGCAGTCGACATATTT |
| 1466A | GCGGCTTTGCCTTCTAATGTTTTAA |
| 1788A | CATCAAATTAATACAGAAGGTCAG |
| cox3 (FP) | ATATGATACTTCTACCGAA |
| cox3 (RP) | CCAGATTATTTCAACAAAA |
| 18S rRNA (FP) | GCTGACTACGTCCCTGCCC |
| 18S rRNA (RP) | ACAATTCATCATATCTTTCAATCGGTA |
| tufA (FP) | GATATTGATTCAGCTCCAGAAGAAA |
| tufA (RP) | ATATCCATTTGTGTGGCTCCTATAA |
