# Supplementary figures and images for: Dual role of an essential HtrA2/Omi protease in the human malaria parasite: Maintenance of mitochondrial homeostasis and induction of apoptosis-like cell death under cellular stress
Source: PLoS Pathog. 2022 Oct 28;18(10):e1010932. doi: 10.1371/journal.ppat.1010932 (PMC9645662; doi:10.1371/journal.ppat.1010932)

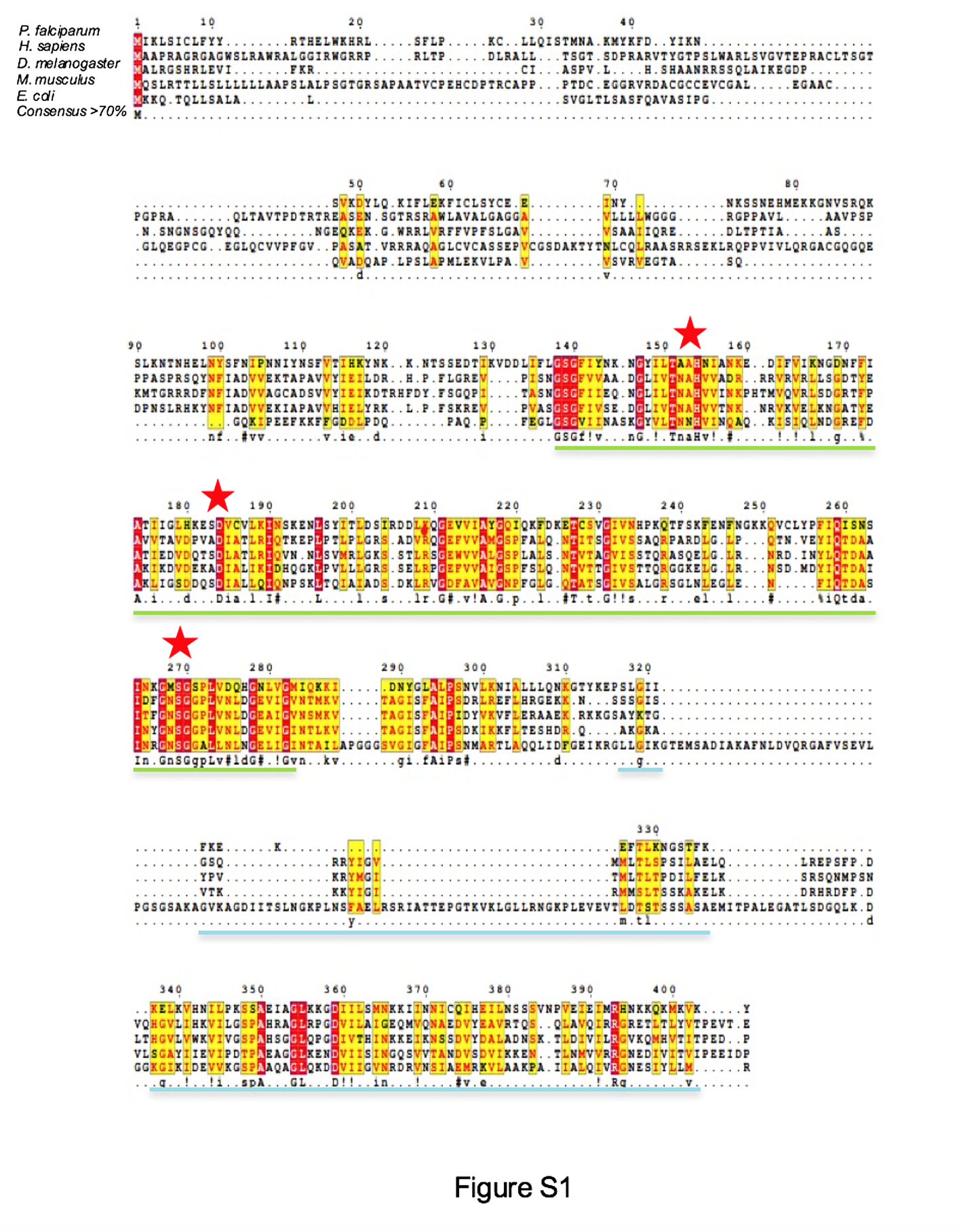

Supplement: S1 Fig — The protease domain is underlined with green line, PDZ domain with blue line, and red asterisks indicate the putative catalytic triad. Amino acids that are identical in all the species are shown in red boxes and amino acids present in at least three of the species are shown in yellow boxes with consensus >70%. In consensus line upper case letters indicates identity and lower case indicates >70% consensus;! indicates I or V; % indicates For Y; # indicates N, D, Q or E;$ indicates L or F. (TIF) [file ppat.1010932.s003.tif]

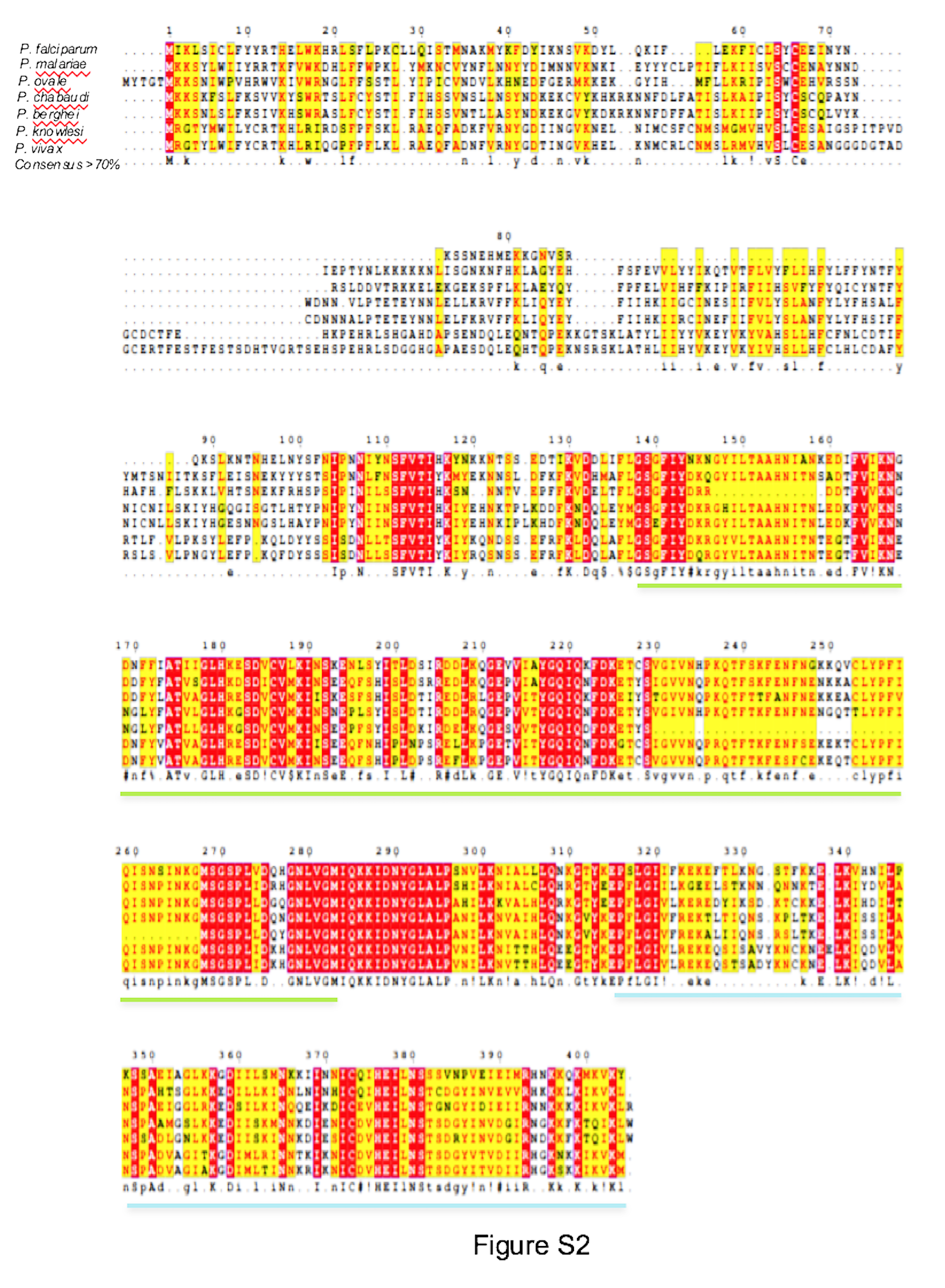

Supplement: S2 Fig — The protease domain is underlined with green line, and PDZ domain is marked with blue line. Amino acids that are identical in all the species are shown in red boxes and amino acids present in at least three of the species are shown in yellow boxes with consensus >70%. In consensus line upper case letters indicates identity and lower case indicates >70% consensus. (TIF) [file ppat.1010932.s004.tif]

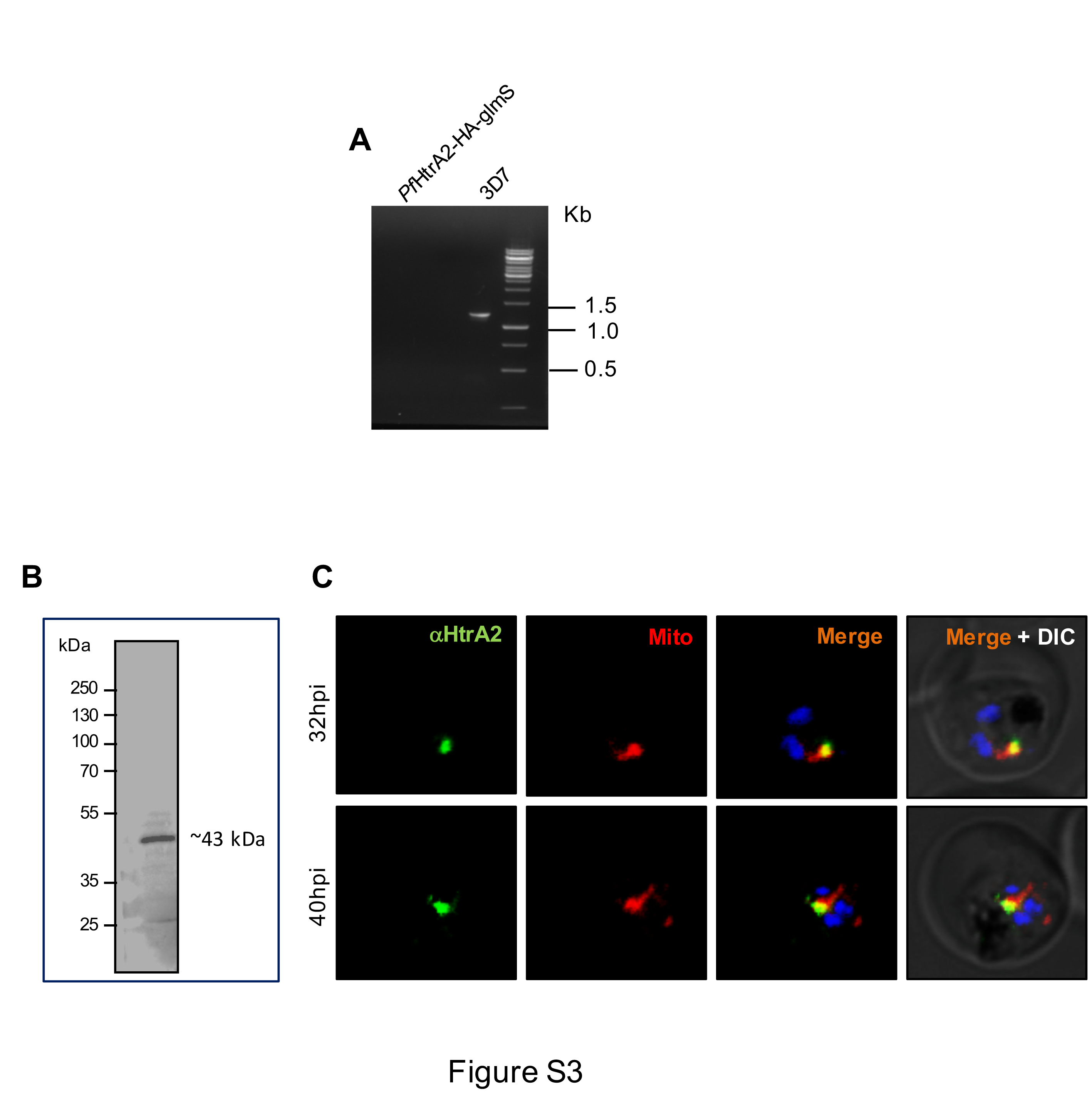

Supplement: S3 Fig — (A) PCR -based analyses using total DNAs of the transgenic parasite culture (purified clonal parasite population) and wild type 3D7 parasite lines, to confirm that the transgenic parasite culture does not contain any wild-type parasites, locations of primers are marked in the schematic (1466A and 1788A). Amplicon of expected size is detected in 3D7 parasite lines but not in the transgenic parasite line. (B-C) Expression and Localization of PfHtrA2 in P. falciparum parasites. (B) Western blot analysis of P. falciparum parasite lysate using anti-PfHtrA antibody (raised against the protease domain) showing detection of full length PfHtrA2 protein of predicted size (~43kDa). (C) fluorescent images of P. falciparum parasite immune-stained using anti-PfHtrA2 antibody and co-stained with MitoTracker stain, PfHtrA2 labelling overlapped with mitochondrial staining; parasite nuclei were stained with DAPI. (TIF) [file ppat.1010932.s005.tif]

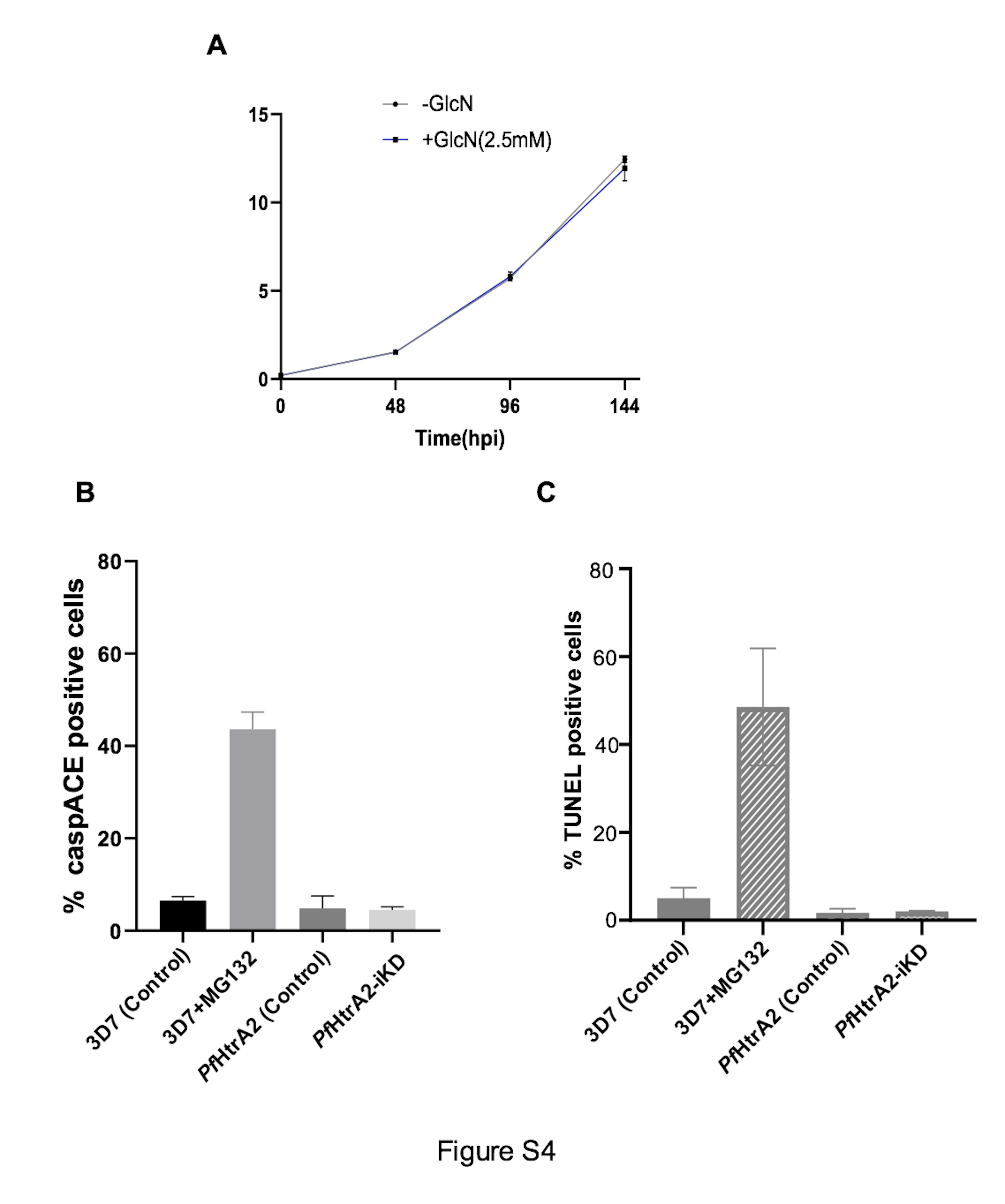

Supplement: S4 Fig — (A) Graph showing wild type 3D7 parasite growth in presence of 2.5 mM glucosamine as compared to solvent control. Tightly synchronized ring-stage parasite culture of parasites were grown with or without glucosamine and their growth was monitored as the formation of new rings determined at 48, 96, and 144hpi; no significant difference in growth was observed between the two sets. (B-C) Inducible knock-down (iKD) of PfHtrA2 (PfHtrA2-iKD) induce non-apoptotic like cell death. Graph showing percentage of CaspACE-labelled (B) and TUNEL positive parasites (C) in the transgenic parasite cultures grown in presence of 2.5 mM glucosamine (PfHtrA2-iKD) and control set; no significant induction of CaspACE or TUNEL labelling was observed in the PfHtrA2-iKD, only basal level of staining was observed as in case of control sets. Wild type 3D7 parasite treated with MG132 or solvent alone were used as positive and negative controls respectively. (TIF) [file ppat.1010932.s006.tif]

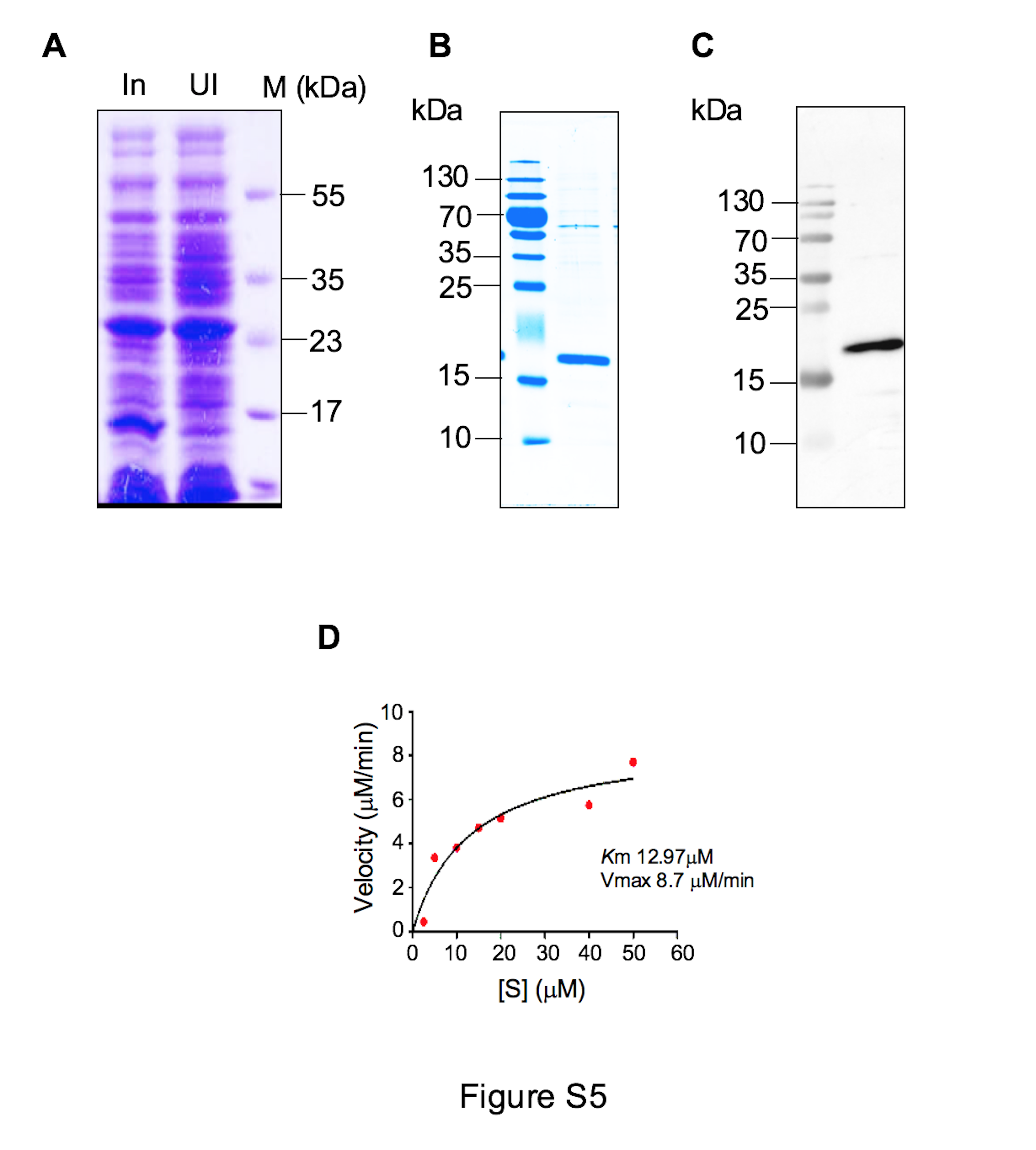

Supplement: S5 Fig — (A) S DS-PAGE gel of E. coli lysate showing induction of expression of recombinant PfHtrA2-protease. (Un: un-induced bacterial culture; In: induced with IPTG). (B) SDS-PAGE gel showing purified recombinant PfHtrA2-protease. (C) Western blot analysis of recombinant PfHtrA2-protease using anti-His antibodies. (D) Graph showing activity of reaction velocity of PfHtrA2-protease activity at various concentrations of substrate used. (TIF) [file ppat.1010932.s007.tif]

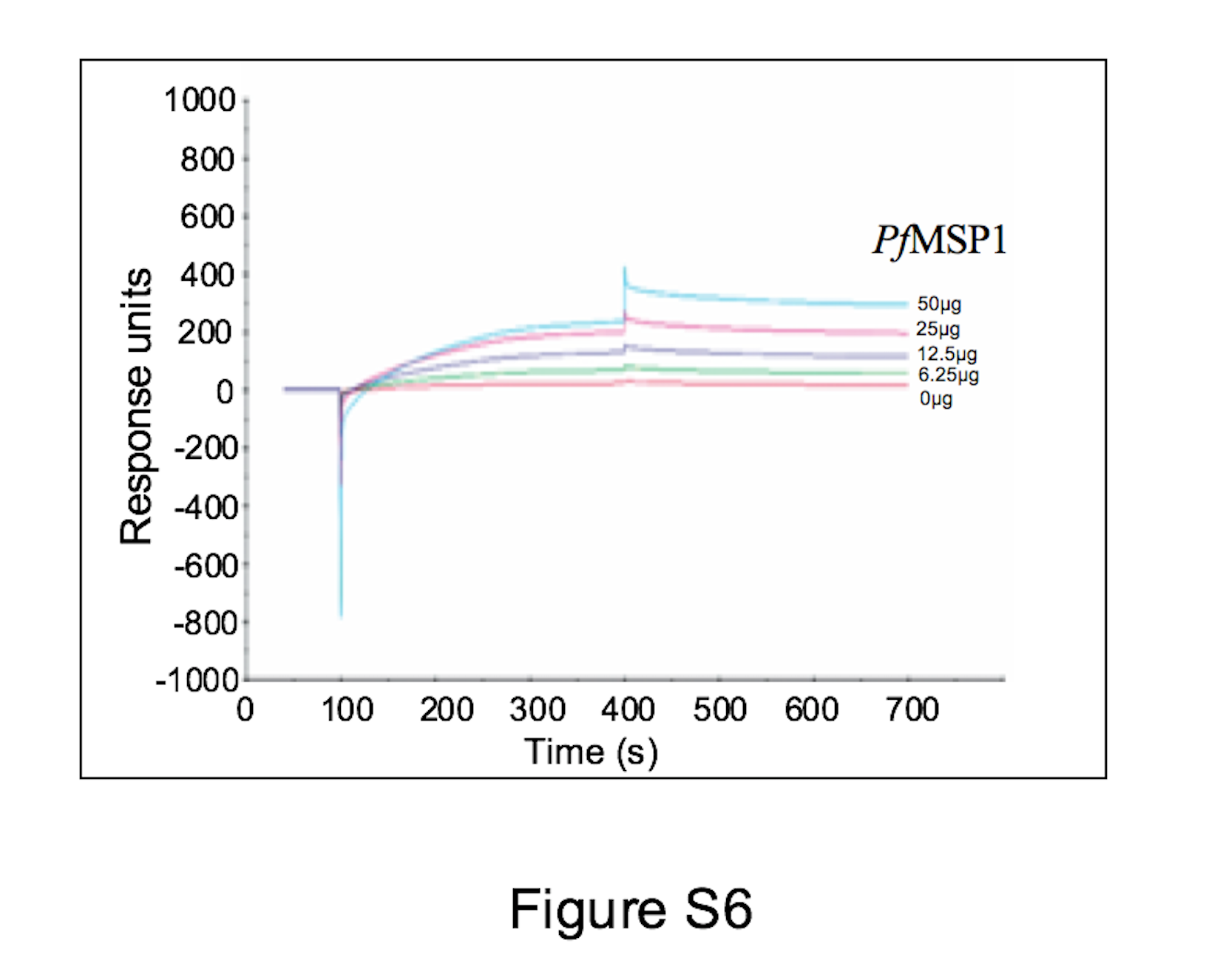

Supplement: S6 Fig — No significant association/dissociation values were obtained at any concentration (TIF) [file ppat.1010932.s008.tif]

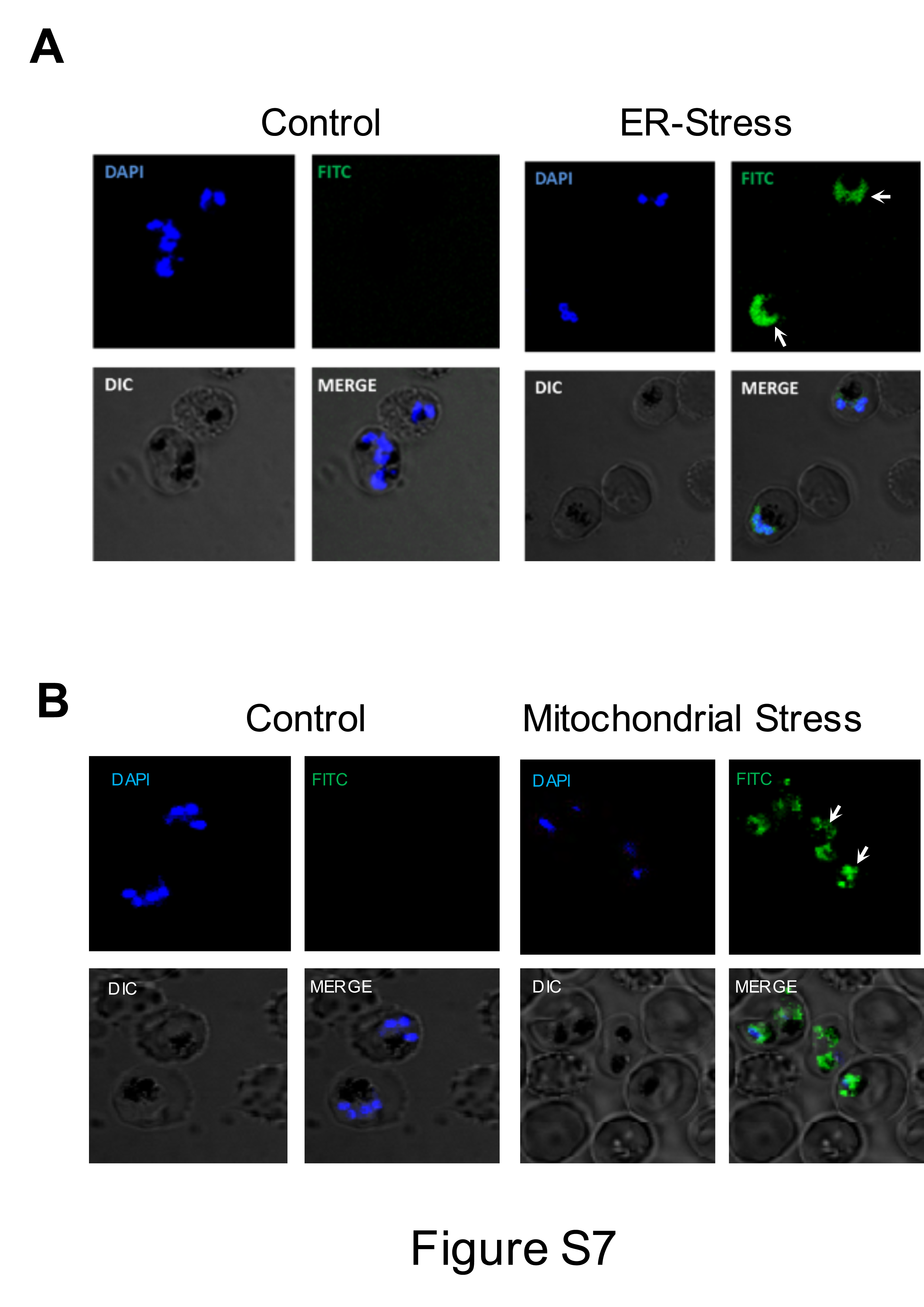

Supplement: S7 Fig — Fluorescent microscopic images showing activation of CaspACE-tagged parasites under ER stress (A) and mitochondrial stress (B), as compared to control. CaspACE stained cells are marked with arrows. (TIF) [file ppat.1010932.s009.tif]
